# Statistical modeling of dynamic eye-tracking experiments: Relative importance of visual stimulus elements for gaze behavior in the multi-group case

**DOI:** 10.3758/s13428-021-01576-8

**Published:** 2021-05-23

**Authors:** Mara Stadler, Philipp Doebler, Barbara Mertins, Renate Delucchi Danhier

**Affiliations:** 1 Department of Statistics, TU Dortmund University, Vogelpothsweg 78, 44227 Dortmund, Germany; 2 Present Address: Department of Statistics, Ludwig Maximilian University of Munich, Ludwigstr. 33, 80539 Munich, Germany; 3 Present Address: Institute of Computational Biology, Deutsches Forschungszentrum für Gesundheit und Umwelt (GmbH), Ingolstaedter Landstr. 1, 85764 Neuherberg, Germany; 4 Department of Culture Studies, TU Dortmund University, Emil-Figge-Str. 50, 44227 Dortmund, Germany

**Keywords:** Eye tracking, Dynamic gaze behavior, Saliency map, Relative importance

## Abstract

This paper presents a model that allows group comparisons of gaze behavior while watching dynamic video stimuli. The model is based on the approach of Coutrot and Guyader (2017) and allows linear combinations of feature maps to form a master saliency map. The feature maps in the model are, for example, the dynamically salient contents of a video stimulus or predetermined areas of interest. The model takes into account temporal aspects of the stimuli, which is a crucial difference to other common models. The multi-group extension of the model introduced here allows to obtain relative importance plots, which visualize the effect of a specific feature of a stimulus on the attention and visual behavior for two or more experimental groups. These plots are interpretable summaries of data with high spatial and temporal resolution. This approach differs from many common methods for comparing gaze behavior between natural groups, which usually only include single-dimensional features such as the duration of fixation on a particular part of the stimulus. The method is illustrated by contrasting a sample of a group of persons with particularly high cognitive abilities (high achievement on IQ tests) with a control group on a psycholinguistic task on the conceptualization of motion events. In the example, we find no substantive differences in relative importance, but more exploratory gaze behavior in the highly gifted group. The code, videos, and eye-tracking data we used for this study are available online.

## Introduction

Exploring gaze behavior is a popular research method in many domains, since it can tell us how we are filtering information and how we might differ in our perception. Examples of between-group comparisons include differences in gaze behavior of experts and laymen (Bernal et al.,, 2014; Giovinco et al.,, 2014; Harezlak, Kasprowski, & Kasprowska, 2018), differences between elderly and younger people (Fontana et al., 2017) or differences in visual exploration due to native language (Stutterheim, Andermann, Carroll, Flecken, & Mertins, 2012). Besides comparisons of natural groups, differences in gaze behavior are studied subject to different experimental conditions like manual driving and highly automated driving (Navarro, Reynaud, & Gabaude, 2017). The data-analytic approach for group comparisons proposed in this paper is illustrated by differences in exploration between a group of people with particularly high cognitive ability and a control group.

Our visual environment is mostly characterized by dynamic processes and therefore the focus in this paper is on modeling dynamic scenes. We model the relative importance (RI) of different stimulus elements in dynamic scenes for gaze behavior for two natural groups by employing raw eye-tracking data. Therefore, we extend the approach of Coutrot and Guyader (2017) to a multi-group case. The model builds on linear combinations of feature maps to form a master saliency map while taking into account the highly dynamic nature of visual exploration, influenced by many time-dependent factors. The feature maps in the model can be, for instance, the static or dynamic salient contents of a video stimulus or predetermined areas of interest (AoIs). In addition, we reflect the individual steps in the modeling process. Before detailing the proposed approach, we review existing techniques for dynamic group comparisons and review modeling based on saliency maps.

### Existing techniques for dynamic group comparisons

There are different approaches for comparing gaze behavior in dynamic scenes. Besides using metrics such as reaction time, dwelling time in AoIs and energy concentration ratios (Bernal et al., 2014; Fontana et al., 2017), some approaches take into account scan patterns. These approaches are based on evaluating similarity with sequence alignment scores followed by testing for statistical differences (Feusner & Lukoff, 2008) or providing a similarity score for two scanpaths based on their morphology and, optionally, duration in an AoI (Frame, Warren, & Maresca 2018). Navarro, Reynaud, and Gabaude (2017) analyze approaches based on the visual screen but without information on displayed images which take into account *x* and *y* axis variability of both groups or detect observer-based AoIs via heat maps and compare the consequent matrices by a Wilcoxon signed-ranks test. Furthermore, Navarro et al., (2017) compare techniques which do take into account the information on the visual stimulus by considering the percentage of time spent looking at a region of 5 degrees around a tangent point or by analyzing gaze positions relative to a dynamic gaze point on the stimulus but with a decomposition of gaze positions in horizontal and vertical components. Coutrot, Hsiao, and Chan (2017) introduce an approach in which hidden Markov models are learned from a group of scanpaths. This is useful to visualize and compare the gaze behavior of two different groups of observers. Other important scanpath algorithms have been introduced by Kübler, Rothe, Schiefer, Rosenstiel, and Kasneci (2017), where the scanpath comparison and classification is based on subsequence frequencies, and by Cristino, Mathot, Theeuwes and Gilchrist (2010), who present an approach for comparing saccadic eye- movement sequences based on the Needleman–Wunsch algorithm used in bioinformatics to compare DNA sequences.

Holmqvist and Andersson (2017) present different over-time calculations, like AoI over time with line graphs showing the proportion of participants gazing at a particular AoI. These methods are illustrated for static stimuli and do not involve direct group comparisons, but also provide feature importance curves that could be compared for different groups. However, this approach is not based on a statistical model, which means that the strength of the effects of the individual features on visual fixations cannot be quantified. Furthermore, this method considers each AoI individually and not in a combined manner. This also leads to multiple allocations of fixations in the case of overlapping AoIs.

### Modeling with saliency maps

Some studies show that saliency maps from the computer vision field play an important role in the prediction of gaze behavior in different settings like watching videos, egocentric vision, or in computer games (Coutrot & Guyader, 2014; Sundstedt, Stavrakis, Wimmer, & Reinhard 2008; Yamada et al.,, 2011). On the other hand, some studies show that tasks overrule saliency when the participant takes the task very seriously (e.g., Chen & Zelinsky, 2006; Land & Hayhoe, 2001). Stimulus-driven saliency, also called bottom-up saliency, can be defined by predetermined AoIs, but also by static and dynamic saliency. Many computational models for visual attention, such as the model by Koch and Ullman (1985), are based on the Feature Integration Theory (FIT) by Treisman and Gelade (1980). A well-known approach that also focuses on features such as contrast, color or orientation, is the model by Itti, Koch, and Niebur (1998). This approach has later been extended by motion filters to obtain saliency models for video stimuli by Peters and Itti (2008). Another saliency model for video stimuli has been proposed by Le Meur, Thoreau, Le Callet, and Barba (2005). Le Meur and Baccino (2012) provide an extensive overview of computational modeling methods of visual attention and survey the strengths and weaknesses of common assessment methods based on diachronic (scanpaths or saliency maps) eye-tracking data. Marat et al., (2008) propose a spatio-temporal saliency model, which is biologically inspired and based on luminance information. In this model, high spatial frequencies are processed to extract luminance orientation and frequency contrast through a bank of Gabor filters and normalized to strengthen the spatially distributed maxima to obtain the static saliency of a frame. Under the assumption of luminance consistency between two consecutive frames, the dynamic pathway of the same model can be used to create dynamic saliency maps. Here, the moving areas are extracted by using low spatial frequencies. This model is also used in the approach of Coutrot and Guyader (2017), on which the method in this work is based.

Coutrot and Guyader (2017) combine the more popular bottom-up features with the observer-based top-down features linearly to a master saliency map. The model takes into account the dynamic aspect of the stimulus by using a statistical shrinkage method, which is a crucial difference from other common models in eye-tracking experiments. The works of Zhao and Koch (2011) and Peters and Itti (2007) for instance, are based on a similar model setup, but use a least-squares approach. Moreover, there exist methods based on deep learning networks that provide even higher correct classification rates and are state-of-the-art in terms of visual saliency prediction (Bylinskii, Isola, Bainbridge, Torralba, & Oliva 2015; Coutrot et al.,, 2017). However, these methods have the disadvantage that they depend on many parameters that are difficult to interpret (Lipton, 2018). In this paper, we extend the approach of Coutrot and Guyader (2017) to a multi-group model, which allows to compare gaze behavior between two or more groups.

The remainder of this paper is organized as follows: After discussing the extension of the approach in Coutrot and Guyader (2017) in the subsequent “Methods”, several worked examples are given in the “Practical application”, followed by concluding remarks in the “Conclusions”.

## Methods

In this section, the idea of modeling eye-position density maps based on the approach of Coutrot and Guyader (2017) is described, though many details differ from the original exposition. The creation of feature maps and the estimation of eye-position density maps based on raw eye-tracking data is reviewed in detail, and the least absolute shrinkage and selection operator (LASSO) is discussed. Subsequently, we present the novel extension of the eye-position density modeling approach to the multi-group case.

### Modeling eye-position density maps

The aim is to predict salient regions in complex natural scenes by linearly combining feature maps to a so-called master saliency map, which identifies regions that might lead to increased visual attention. The features in the model can refer to the stimulus (like contrast, motion, or predetermined AoIs), the so-called bottom-up features, or to the observer, the top-down features (like group membership). An often-observed behavior-based bias is the center bias (e.g., Tseng, Carmi, Cameron, Munoz, & Itti, 2009). This top-down feature describes the tendency to visually focus rather on the center than on the edges of a stimulus. The weights of the feature maps in the model vary systematically over time. The choice of feature maps also plays an important role since it has a strong impact on the predictions quality.

Let ***S*** be a master saliency map, ***M***_*k*_(*t*) the feature map for the *k* th feature at time *t*, *k* ∈{1,...,*K*}, and *β*_*k*_(*t*) the corresponding feature map weight at time *t*. The master saliency map ***S***(*t*) is given by the linear combination

$$ \begin{array}{@{}rcl@{}} \boldsymbol{S}(t) = \sum\limits_{k=1}^{K} \beta_{k}(t) \boldsymbol{M}_{k}(t). \end{array} $$

We suppress the dependency on time in the notation for ease of exposition, i.e., ***S*** = ***S***(*t*), ***M***_*k*_ = ***M***_*k*_(*t*) and *β*_*k*_ = *β*_*k*_(*t*) for all *k*. The maps ***S*** and ***M***_*k*_, *k* = 1,...,*K*, can be understood as vectors with a length corresponding to the number of pixels of the stimulus frame. The vector of weights ***β*** is learned using eye-tracking data. Visual experiments are dynamic processes affected by many time-related factors, so the statistical shrinkage method LASSO is used to sieve out relevant feature maps.

### Feature map generation

The generation of feature maps to be included in the model is described next. Note that all feature maps are represented by matrices, and all are normalized to obtain a bivariate probability density function by dividing each entry through the sum of all entries of the map.

#### Uniform map

The uniform map is a bottom-up feature with the same value $\frac {1}{w \cdot h}$ at each entry or pixel, $\boldsymbol {M}_{U}=(\frac {1}{w \cdot h})_{i=1,...,w,~j=1,..,h} \in \mathbb {R}^{w \times h}$, where *w* and *h* represent the stimulus width and the stimulus height in pixels. This feature represents a “catch-all hypothesis” for fixations, which can only be weakly explained by the other features.

#### Center bias map

The center bias is a bottom-up feature generated by a time-independent bivariate Gaussian function $\mathcal {N}(0,\boldsymbol {\Sigma })$ with a diagonal covariance matrix $\boldsymbol {\Sigma } = \text {diag}(\sigma _{x}^{2},$ $ {\sigma _{y}^{2}})$, which is centered at the center of the monitor. Standard deviations *σ*_*x*_ and *σ*_*y*_ are chosen proportional to frame size by dividing the stimulus height and width by 12.

#### Static and dynamic saliency map

The static and dynamic saliency maps are top-down features that highlight areas of the stimulus that stand out statically or dynamically from the other areas of the stimulus. Saliency maps can be created using different saliency models. In this paper, saliency maps are first determined using two different saliency models and then the resulting feature map weights are compared. The Graph-Based Visual Saliency Algorithm (GBVS) and the Real-Time Three-Path Saliency Model (TVSM) are used for this purpose. The comparison of these rather different approaches is carried out to get an impression of the influence of the choice of the saliency model on the results in the eye-position density map modeling approach. The GBVS algorithm is a graph-based approach (Harel, Koch, & Perona, 2006). The spatio-temporal saliency model TVSM is a biologically inspired model based on luminance information (Marat, Rahman, Pellerin, Guyader, & Houzet, 2013) and is also applied in Coutrot and Guyader (2017). Both approaches use the same model to create dynamic saliency maps and static saliency maps. The approach to model dynamic saliency maps considers information from the previous frame. For this reason, it is not possible to create a dynamic saliency map for the first frame.

We find that for stimuli where the dynamic content is also statically very different from the remainder of the stimulus, the TVSM algorithm is more suitable for determining the saliency maps: Fig. 9 includes the comparison of the GVBS and the TVSM saliency maps for an illustrative frame as well as some resulting estimated curves, which show that the resulting feature map weights for the video stimulus differ considerably between the approaches. The contrary course of the feature map weightings of static and dynamic saliency due to the correlation of the two features is particularly strong under the GBVS approach. For dynamic scenes where the statically salient content is more distinct from the dynamically prominent content, such as the stimulus from the freely accessible supplementary material from Coutrot and Guyader (2017), the inaccuracy of the GBVS algorithm has little effect on the estimated feature map weights.

**Fig. 1:**
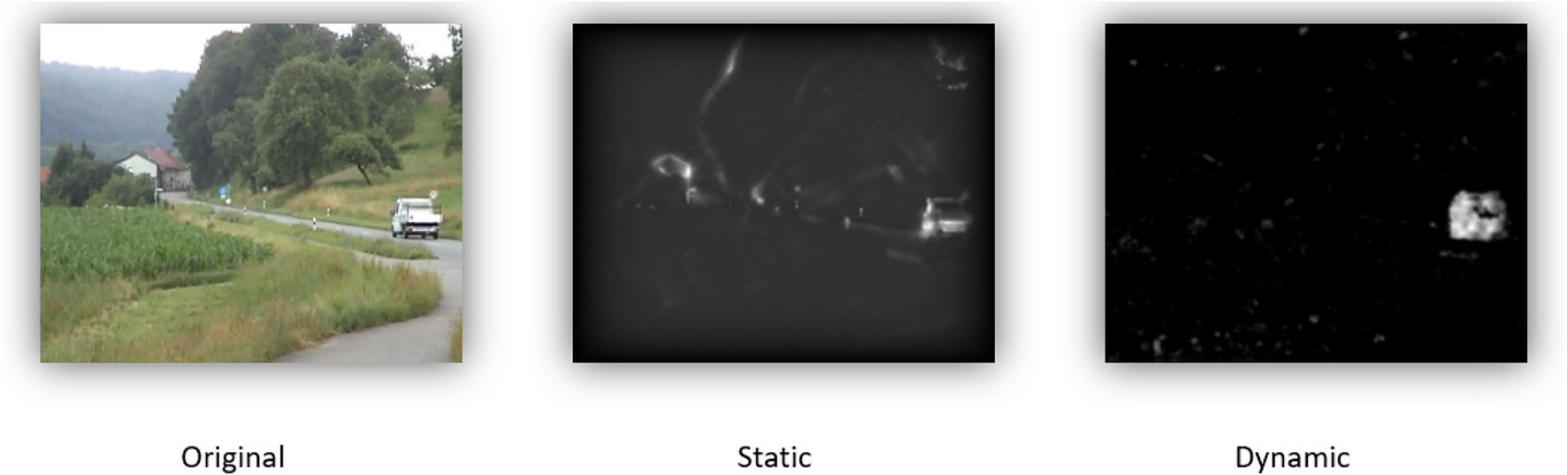
Frame of a video stimulus (*left*) with corresponding static (*middle*) and dynamic (*right*) saliency maps calculated with TVSM. Contrasts and luminance influence the static map, while the moving truck in the otherwise steady scene dominates the dynamic salience

Consequently, the saliency features are determined using the TVSM algorithm. Figure 1 shows the static and dynamic saliency map as well as the original frame for one frame of the video stimulus *car cornfield* from our experiment.

#### Areas of interest (AoIs)

**Fig. 2:**
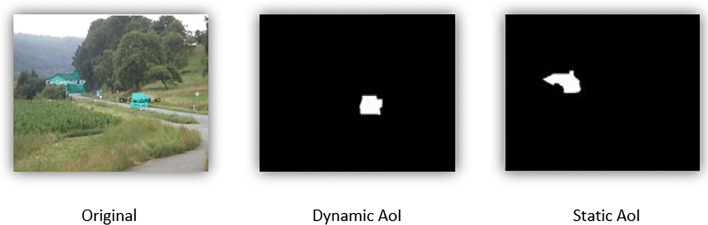
Frame of a video stimulus with highlighted AoIs (*left*) and dynamic (*middle*) and static AoI maps (*right*) corresponding to binary matrices. The dynamic AoI map differs from frame to frame while the static AoI map is the same for each frame of the video stimulus since it does not move and there is no camera motion

The AoIs are defined as polygons with known vertices. Based on these coordinates, we create binary matrices for each frame, which are 1 on the pixels inside the polygon and 0 otherwise. Figure 2 illustrates the dynamic and static AoI in a video stimulus.

### Eye-position density map estimation

For each natural or experimental group, an eye-position density map is estimated frame by frame from raw eye-tracking data by kernel density estimation, a non-parametric approach for estimation of probability densities. Since the stimuli are two-dimensional videos, a bivariate kernel density with bivariate least squares cross-validation bandwidth matrix is appropriate (e.g., Duong, 2004). The bivariate kernel density estimator on a grid out of pixels ***x*** for a random sample out of *N* fixation coordinates ***X***_1_,...,***X***_*N*_ is given by

$$ \begin{array}{@{}rcl@{}} \hat{f}(\boldsymbol{x}; \boldsymbol{H}) = N^{-1} \sum\limits_{i=1}^{N} K_{\boldsymbol{H}}(\boldsymbol{x}-\boldsymbol{X}_{i}), \end{array} $$

with two-dimensional vectors ***x*** = (*x*_1_,*x*_2_)^*T*^ and ***X***_*i*_ = (*X*_*i*1_,*X*_*i*2_)^*T*^,*i* = 1,...,*N*. The scaled kernel is denoted by *K*_***H***_ and $\boldsymbol {H} \in \mathbb {R}^{2\times 2}$ is a non-random, symmetric, positive definite bandwidth matrix. The relation to the non-scaled kernel *K* is given in a general form by $K_{\boldsymbol {H}}(\boldsymbol {x})=|\boldsymbol {H}|^{-1/2}K(\boldsymbol {H}^{-1/2}\boldsymbol {x})$. The scaled bivariate gaussian kernel, on which the calculations in this paper are based, is given by $K_{\boldsymbol {H}}(\boldsymbol {x})=(2\pi )^{-1}|\boldsymbol {H}|^{-1/2} \exp (-\frac {1}{2}\boldsymbol {x}^{T} \boldsymbol {H}^{-1}\boldsymbol {x})$.

The bandwidth matrix $\hat {\boldsymbol {H}}_{\text {LSCV}}$ is the solution of the minimization problem argmin_***H***_LSCV(***H***), with

$$ \begin{array}{@{}rcl@{}} \text{LSCV}(\boldsymbol{H}) = {\int}_{\mathbb{R}^{d}} \hat{f}(\boldsymbol{x}; \boldsymbol{H})^{2} d\boldsymbol{x} - 2 N^{-1} \sum\limits_{i=1}^{N} \hat{f}_{-i}(\boldsymbol{X}_{i}; \boldsymbol{H}), \end{array} $$

where $\hat {f}_{-i}(\boldsymbol {X}_{i}; \boldsymbol {H})=(N-1)^{-1} {\sum }_{j=1, j\ne i}^{N} K_{\boldsymbol {H}}(\boldsymbol {X}_{i}-\boldsymbol {X}_{j})$ is the *leave-one-out*-estimator. This procedure differs from the kernel density estimation in the work of Coutrot and Guyader (2017), in which a bivariate Gaussian kernel with a standard deviation of 1 degree of visual angle is chosen. In particular, a new bandwidth is selected here for each frame, which allows for more variability between the densities of the individual frames (a comparison of the two approaches can be seen in Fig. 8). A smooth eye position density map ***Y*** results. With this approach, no further background information on the experimental setup (like visual angle) is required. Overall, the smoother the kernel density estimation, the less individual feature maps stand out and the more similar the resulting weights are.

### Least absolute shrinkage and selection operator algorithm

The advantage of the LASSO over other regression methods, especially least squares regression or the expectation maximization algorithm, is that it allows selecting relevant features and to reject the other features. This property can lead to a more efficient and more interpretable model (Hastie, Tibshirani, & Friedman, 2009). Here, the LASSO shrinks feature map weights ***β*** by imposing a *L*_1_ penalty with irrelevant map weights shrunk to 0 and, hence, removed entirely from the master saliency map.

Recall that the eye-position density map is denoted by ***Y***, and that the master saliency is equal to ${\sum }_{k=1}^{K} \beta _{k} \boldsymbol {M}_{k}$. The parameter *λ* > 0 is a tuning parameter, which controls the amount of shrinkage. The LASSO estimate solves the minimization problem

$$ \begin{array}{@{}rcl@{}} {\boldsymbol{\beta}}^{LASSO}(\lambda)=\text{argmin}_{\boldsymbol{\beta}} \left\{ (\boldsymbol{Y}-\sum\limits_{k=1}^{K} \beta_{k} \boldsymbol{M}_{k})^{2} + \lambda \sum\limits_{k=1}^{K}|\beta_{k}| \right\}. \end{array} $$

For *λ* = 0 the LASSO algorithm corresponds to the least squares estimate. For $\lambda \rightarrow \infty $ the weights *β*_*k*_, *k* = 1,...,*K*, are shrunk towards zero. For an increasing *λ*, the variance decreases and the bias increases (James, Witten, Hastie, & Tibshirani, 2013).

The R package *glmnet* (Friedman, Hastie & Tibshirani, 2010) finds ***β***^*LASSO*^(*λ*) values for a regularization path, i.e., for a sequence of *λ* values. Following Coutrot and Guyader (2017), *λ* is chosen so that ***β***^*LASSO*^(*λ*) is optimal in terms of the Bayesian Information Criterion (BIC), given here by

$$ \begin{array}{@{}rcl@{}} \text{BIC} = \text{BIC}(\boldsymbol{S}|\boldsymbol{Y}) = -2\text{~log~}L(\boldsymbol{S}|\boldsymbol{Y}) + K \text{~log~} n, \end{array} $$

where *L* is Gaussian likelihood of ***S*** = ***S***(*λ*), *K* is the number of feature maps in the model (equal to the number of nonzero *β*_*k*_) and *n* = *w* ⋅ *h* denotes the number of pixels in ***Y***. Zou, Hastie, and Tibshirani (2007) show that the number of non-zero coefficients provides an unbiased estimate of the degrees of freedom in LASSO, which does not require further assumptions on the predictors. In addition, it is shown that the unbiased estimator is asymptotically consistent and thus model selection criteria, such as the BIC, are acceptable.

### Extension to multi-group case

The extension of the approach of Coutrot and Guyader (2017) is based on the method for modeling interactions between qualitative and quantitative predictors in general linear models, e.g., Kutner, Nachtsheim, and Neter (2005). To extend the model for the two-group case, a binary dummy variable $\boldsymbol {\tilde {M}}_{G}$ is introduced, which denotes whether the information on the *j* th pixel refers to the treatment or the control group and is given by

$$ \boldsymbol{\tilde{M}}_{Gj} = \begin{cases} 1, \text{ if } j~ \text{refers to the treatment group} \\ 0, \text{ if } j~ \text{refers to the control group}, \end{cases} $$

where *j* = 1,...,2 ⋅ *w* ⋅ *h*, with *w* the number of pixels in width and *h* the number of pixels in height. In the following model, extension of the first *w* ⋅ *h* entries refer to the treatment group and the second *w* ⋅ *h* entries refer to the control group. Thus, $\boldsymbol {\tilde {M}}_{G}$ is given by $\boldsymbol {\tilde {M}}_{G} := \left [\boldsymbol {1}_{w\cdot h}^{T} \boldsymbol {0}_{w\cdot h}^{T}\right ]^{T} \in \mathbb {R}^{2 \cdot w \cdot h}$.

The feature maps are given in form of vectors in the model and the variable $\boldsymbol {\tilde {M}}_{G}$ is interacted with each feature map in the model. This is done by elementwise vector multiplication, denoted by “∘”. The model with *K* feature maps ***M***_1_,...,***M***_*K*_ is given by

1 $$ \begin{array}{@{}rcl@{}} \boldsymbol{\tilde{Y}}=\boldsymbol{\beta} \boldsymbol{\tilde{M}}+\epsilon, \end{array} $$

where $\boldsymbol {\tilde {M}}=\Bigl [\boldsymbol {\tilde {M}}_{1} ~ ...~ \boldsymbol {\tilde {M}}_{K} ~~\boldsymbol {\tilde {M}}_{1}\circ \boldsymbol {\tilde {M}}_{G} ~...~ \boldsymbol {\tilde {M}}_{K} \circ \boldsymbol {\tilde {M}}_{G} \Bigr ]$ denotes the design matrix with $\boldsymbol {\tilde {M}}_{i} := \left [ \boldsymbol {M}_{i}^{T} \boldsymbol {M}_{i}^{T}\right ]^{T}$ $\in \mathbb {R}^{2 \cdot w \cdot h}$, for *i* = 1,...,*K*, $\boldsymbol {\tilde {Y}} = \left [\boldsymbol {Y}_{T}^{T} \boldsymbol {Y}_{C}^{T}\right ]^{T} \in \mathbb {R}^{2 \cdot w \cdot h}$ with ***Y***_*T*_ and ***Y***_*C*_ the eye-position density maps of the treatment and control group in form of a vector and $\boldsymbol {\beta }=(\beta _{1},..., \beta _{K},\beta _{1,G},...,\beta _{K,G})^{T} \in \mathbb {R}^{2 \cdot K}$ the regression coefficient vector. The density on the *j* th pixel is therefore given by

$$ \begin{array}{@{}rcl@{}} \boldsymbol{\tilde{Y}}_{j} & = & \beta_{1} \boldsymbol{\tilde{M}}_{1j} +...+ \beta_{K} \boldsymbol{\tilde{M}}_{Kj} + \beta_{1,G} (\boldsymbol{\tilde{M}}_{1j} \cdot \boldsymbol{\tilde{M}}_{Gj}) + ...\\&&+ \beta_{K,G} (\boldsymbol{\tilde{M}}_{Kj} \cdot \boldsymbol{\tilde{M}}_{Gj}) + \epsilon_{j} \\ & = & \begin{cases}(\beta_{1} + \beta_{1,G}) \boldsymbol{\tilde{M}}_{1j} + ...+(\beta_{K} + \beta_{K,G}) \boldsymbol{\tilde{M}}_{Kj} + \\ ~~~... + \epsilon_{j}, j = 1,...,n\\ \beta_{1} \boldsymbol{\tilde{M}}_{1j} + ... + \beta_{K} \boldsymbol{\tilde{M}}_{Kj} + \epsilon_{j}, j = n + 1,..., 2 \cdot n, \end{cases} \end{array} $$

with *n* = *w* ⋅ *h*. If *β*_1,*G*_,...,*β*_*K*− 1,*G*_ or *β*_*K*,*G*_ differ significantly from zero, it can be interpreted as differences in gaze behavior between the two groups. Confidence intervals therefore need to be estimated, which will not be further specified in this paper.

## Practical application

In the following section, we describe the structure of the experiment, the stimulus material, as well as the available data material and data processing. Subsequently, the method is applied to the stimulus and data material. In addition to the analysis of the stimuli in our specific experiment, we also include the evaluation of two static stimuli in the Appendix to demonstrate that our method also works for other types of stimuli. The analyses are performed in the statistics software R (R Core Team, 2020). The code, visual material, and eye-tracking data we used for this study are available online.^1^

### Material

#### Participants and experiment

The eye-tracking experiment was carried out with two groups at different time points. The first group consists of *N*_*T*_ = 33 members of the *Mensa in Deutschland e.V.*, an association for participants with particularly high cognitive abilities. This group is denoted as the treatment group, where a particularly high cognitive ability stands for the treatment. The second group contains *N*_*C*_ = 102 participants, which are primarily members of a bachelor’s program in German philology. This group is considered to be the control group. All participants are multi-lingual and speak German at a native-speaker level. The treatment group in our experiment was aware that their gaze behavior would be compared with a control group, which could influence their gaze behavior.

In the experiment, several video stimuli are presented to the participants. The task was to briefly describe orally what is happening in the video. This task (or pseudo-task) is common in many psycholinguistic gaze behavior studies since it aims to achieve greater comparability between participants, as visual behavior can vary greatly without any task (e.g., Castelhano, Mack, & Henderson, 2009).

#### Visual material

The video stimuli are taken from a study that compares the gaze behavior of speakers of different native languages (Stutterheim, Andermann, Carroll, Flecken, & Mertins, 2012). A distinction was made between speakers of an aspect language and non-aspect language. In terms of the use of tenses, aspect languages, such as English, distinguish between an ongoing action (John was crossing the street) and a completed action in the past (John has crossed the street). Non-aspect languages, such as German, do not make such a distinction, but need time adverbs to clarify that an action is happening right now. In the study, it was shown that speakers of non-aspect languages, when considering dynamic stimuli, put a stronger focus on the expected—but not occurring—endpoint towards which an object is moving (Stutterheim et al., 2012). In the context of the current work, influence of cognitive ability on gaze behavior is studied, while keeping the variable language constant. In a study by Vigneau, Caissie, and Bors (2006) on differences in gaze behavior when solving the Advanced Progressive Matrices Test, a speech-free multiple-choice intelligence test, it could be shown, for example, that subjects with high test scores consider all elements of the matrix to be completed. In contrast, subjects with low test scores only considered the elements in the row and column of the element to be completed in the matrix.

The video stimuli contain one moving object, the dynamic AoI, and we have defined a fixed end point, the static AoI. The stimuli end before the end point is reached by the moving object. The stimuli have no camera pan and no sound. The procedure is exemplified on several stimuli in this paper and the detailed procedure is described using the stimulus *car cornfield* as an example.

**Fig. 3:**
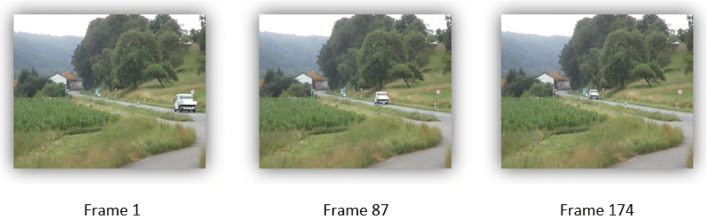
Excerpt of the video stimulus at the beginning, in the middle, and at the end (1st, 87th, and 174th frame)

The refresh rate is 25 Hz and the resolution of the stimulus is *w* ⋅ *h* = 720 × 576 pixels. This video stimulus shows a car, the dynamic AoI, driving in the direction of a house, representing the static AoI, see Fig. 3. The duration of the video stimulus is approximately 7 s and therefore the stimulus consists out of *N*_*F*_ = 174 frames.

#### Eye-tracking data

The eye-tracking data are given as *x* and *y* coordinates of fixations and saccades on the monitor. Following Coutrot and Guyader (2017), only the coordinates of the right eye are considered. The data were recorded with an SMI RED 60 device. The distance of a participant to the monitor was between 55 and 65 cm. The resolution of the monitor is 1920 × 1080 pixels and the stimulus was enlarged to full monitor height and proportionally adjusted in width. Therefore, the video area has a resolution of 1350 × 1080 pixels with black areas on the sides with a width of 258 pixels each. The fixations and saccades were recorded at a sampling rate of 60 Hz. The upper left corner of the monitor represents the coordinate (0,0), which is also recorded if there is a loss of vision or if the respondent blinks.

#### Data processing

The number of recorded fixations or saccades varies slightly between 407 and 410 data points per participant in the treatment group and between 407 and 419 in the control group due to eye-tracker inaccuracies. The dataset does not provide any information about the points in time at which the gaze coordinates are lost, which is why the number of gaze coordinates is shortened by discarding the last gaze coordinates to the minimum available number of gaze coordinates per respondent. Thus, inaccuracies of up to 12/60 s can be assumed. The gaze coordinates (0,0) are removed, as they represent that the gaze could not be tracked. In addition, the coordinates tracked outside the stimulus area on the monitor are removed. The tracking rate of 60 Hz and the refresh rate of 25 Hz result in 2.4 coordinates per person and frame. In order to consider one coordinate per person per frame, the first viewing coordinate that remains completely on the respective frame is selected. With 2.4 view coordinates per frame, the 1st, 4th, 6th, 9th, 11th, 13th etc. are thus selected. The remaining view coordinates are not included in the analysis.

When estimating causal effects in observational data, a randomized experiment should be replicated as accurately as possible to ensure that the distribution of covariates in the treatment and control groups is as similar as possible. To ensure this, a matching is carried out. Since the groups have rather small overlaps in their covariates, a propensity score matching (PSM) with an optimal matching algorithm and subsequent balance diagnostics (Zhang, Kim, Lonjon, & Zhu, 2019) on the covariates gender and age is performed using the R package *MatchIt* (Ho, Imai, King, & Stuart, 2011). PSM can be helpful if there is a high level of imbalance in the covariates (King & Nielsen, 2019). By using a caliper of 0.1 the matching algorithm selects only 25 participants from the control group and ten participants from the extreme group with high cognitive abilities and thus rejects 77 participants from the control group and 23 participants from the group of participants with high cognitive abilities. Although the result does not provide satisfactory group sizes, the two-group model is illustrated on the basis of these matched groups.

### Results

**Fig. 4:**
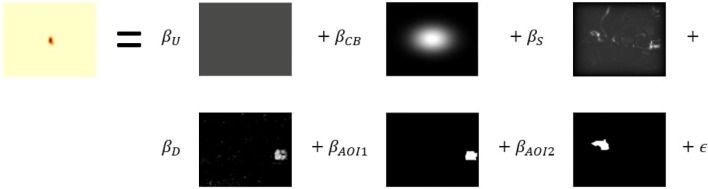
Model illustration for the second frame of the video stimulus (U = uniform, CB = center bias, S = static, D = dynamic)

Figure 4 illustrates the single-group model for one frame of the video stimulus. The two-dimensional maps can be understood as matrices ***M*** of the dimension *w* × *h*, where *w* stands for the number of pixels in width and *h* for the number of pixels in height of the stimulus. Each pixel corresponds to a number on the grayscale, where 0 stands for black and 1 for white. The uniform (U), center bias (CB), static saliency (S), and dynamic saliency (D) maps, as well as the dynamic AoI (AOI1) and the static AoI (AOI2) are all included in the model. The matrices are treated as vectors in the model definition, so that the value of the kernel density estimate on a pixel corresponds to an observation in the model. Therefore, $\boldsymbol {M}_{i}:=\vec {\boldsymbol {M}}_{i}$, *i* ∈{U, CB, S, D, AOI1, AOI2}, applies. Mathematically, the model has the following form,

$$ \begin{array}{@{}rcl@{}} \boldsymbol{M} &=& \beta_{U} \boldsymbol{M}_{U} + \beta_{CB} \boldsymbol{M}_{CB} + \beta_{S} \boldsymbol{M}_{S} + \beta_{D} \boldsymbol{M}_{D} \\ &&+ \beta_{AOI1} \boldsymbol{M}_{AOI1} + \beta_{AOI2} \boldsymbol{M}_{AOI2} + \epsilon, \end{array} $$

where $\boldsymbol {Y}, \boldsymbol {M}_{i} \in \mathbb {R}^{w\cdot h}$, *i* ∈{U, CB, S, D, AOI1, AOI2}, and *w* ⋅ *h* = 576 ⋅ 720 = 414720. The residuals *𝜖* ignore any remaining spatial dependencies and framewise homoscedasticity is assumed. Each feature map vector is divided by the sum of all entries of the vector to obtain probability density functions. The eye-position density map and feature maps are centered and standardized in the model. Thus, the units on the *y*-axis are standard deviations.

**Fig. 5:**
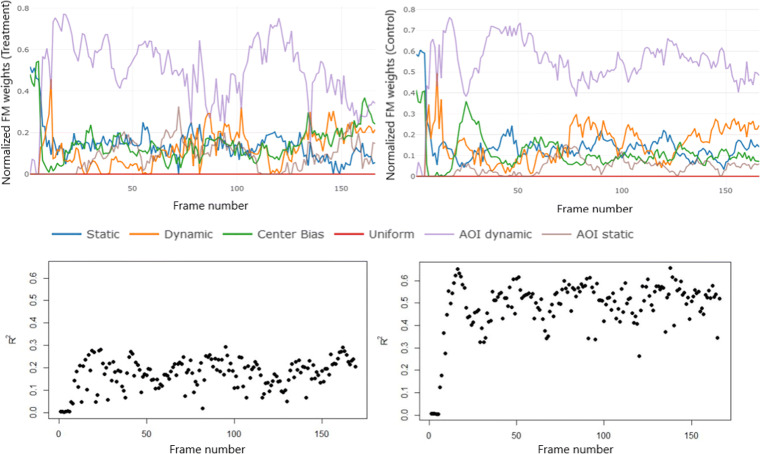
RI curves of respective feature for both groups separately in the non-extended model (*top*) and *R*^2^ values for both groups (*bottom*) (*left*: treatments, *right*: controls)

First, the initial model is adapted separately for both unmatched groups of participants. The following Fig. 5 shows the estimated relative importance (RI) curves and the adjusted coefficient of determination *R*^2^ for each frame. The term ‘relative importance’ refers to the effect of each feature map on the prediction of the fixation density on the corresponding frame in the stimulus compared to the effect of the other feature maps in the model. The RI of the feature maps can be compared between different feature maps on one frame (at the same time) or between several frames (throughout the stimulus duration).

In both groups, the feature maps do not predict the fixations on the first frames very well. After about the 10th frame, the *R*^2^ values increase. Since it is assumed that there are latent influences on human gaze behavior, even lower *R*^2^ values can be considered acceptable. The curve of the coefficient of determination *R*^2^ of the treatment group is similar to the curve of the control group, but on a noticeably lower level. It can be concluded that the participants of the treatment group exhibit a more explorative behavior during this stimulus and thus the feature maps predict the coordinates of the fixations less accurately. This finding is also obtained when drawing a random sample from the control group that corresponds to the sample size of the treatment group.

The extension to a two-group model for the *j* th pixel, *j* = 1,...,2 ⋅ *w* ⋅ *h* = 2 ⋅ 576 ⋅ 720 = 829440, is given by

$$ \begin{array}{@{}rcl@{}} \boldsymbol{\tilde{Y}}_{j} & = & \beta_{U} \boldsymbol{\tilde{M}}_{Uj} + \beta_{CB} \boldsymbol{\tilde{M}}_{CBj} + \beta_{S} \boldsymbol{\tilde{M}}_{Sj} + \beta_{D} \boldsymbol{\tilde{M}}_{Dj}  \\ & &+ \beta_{AOI1} \boldsymbol{\tilde{M}}_{AOI1j}+\beta_{AOI2} \boldsymbol{\tilde{M}}_{AOI2j} \\&&+ \beta_{U,G} (\boldsymbol{\tilde{M}}_{Uj} \cdot \boldsymbol{\tilde{M}}_{Gj}) \\ & & + \beta_{CB,G} (\boldsymbol{\tilde{M}}_{CBj}\cdot \boldsymbol{\tilde{M}}_{Gj})+\beta_{S,G} (\boldsymbol{\tilde{M}}_{Sj} \cdot \boldsymbol{\tilde{M}}_{Gj}) \\ & & + \beta_{D,G} (\boldsymbol{\tilde{M}}_{Dj} \cdot \boldsymbol{\tilde{M}}_{Gj}) + \beta_{AOI1,G} (\boldsymbol{\tilde{M}}_{AOI1j} \cdot \boldsymbol{\tilde{M}}_{Gj}) \\ &&+\beta_{AOI2,G} (\boldsymbol{\tilde{M}}_{AOI2j} \cdot \boldsymbol{\tilde{M}}_{Gj}) + \epsilon_{j},\\ & = & \begin{cases} (\beta_{U} + \beta_{U,G}) \boldsymbol{\tilde{M}}_{Uj} + (\beta_{CB} + \beta_{CB,G}) \boldsymbol{\tilde{M}}_{CBj} + ... \\+ \epsilon_{j}, \text{ for } j = 1,..., n\\ \beta_{U} \boldsymbol{\tilde{M}}_{Uj} + \beta_{CB} \boldsymbol{\tilde{M}}_{CBj} + ... + \epsilon_{j}, \text{ for } j = n+1,\\...,2 \cdot n, \end{cases} \end{array} $$

with $\boldsymbol {\tilde {M}}_{i} := \left [ \boldsymbol {M}_{i}^{T} \boldsymbol {M}_{i}^{T}\right ]^{T}$ $\in \mathbb {R}^{2 \cdot w \cdot h}$ for *i* ∈{U, CB, S, D, AOI1, AOI2}, $\boldsymbol {\tilde {M}}_{G} := \left [ \boldsymbol {M}_{G,Tre}^{T} \boldsymbol {M}_{G,Con}^{T}\right ]^{T}= \left [\boldsymbol {1}_{w\cdot h}^{T}\boldsymbol {0}_{w\cdot h}^{T}\right ]^{T}$ $\in \mathbb {R}^{2 \cdot w \cdot h}$ and *n* = *w* ⋅ *h* = 414720.

The bivariate kernel density estimate $\boldsymbol {\tilde {Y}} = \left [\boldsymbol {Y}_{Tre}^{T} \boldsymbol {Y}_{Con}^{T}\right ]^{T}$ $\in \mathbb {R}^{2 \cdot w \cdot h}$ and the feature maps are centered and standardized separately for each group. The kernel density estimation in the groups is carried out separately for both groups and is therefore based on different bandwidths. A standardization across both groups could lead to a group having a strong peak if there are strong mean differences in the densities of the groups.

**Fig. 6:**
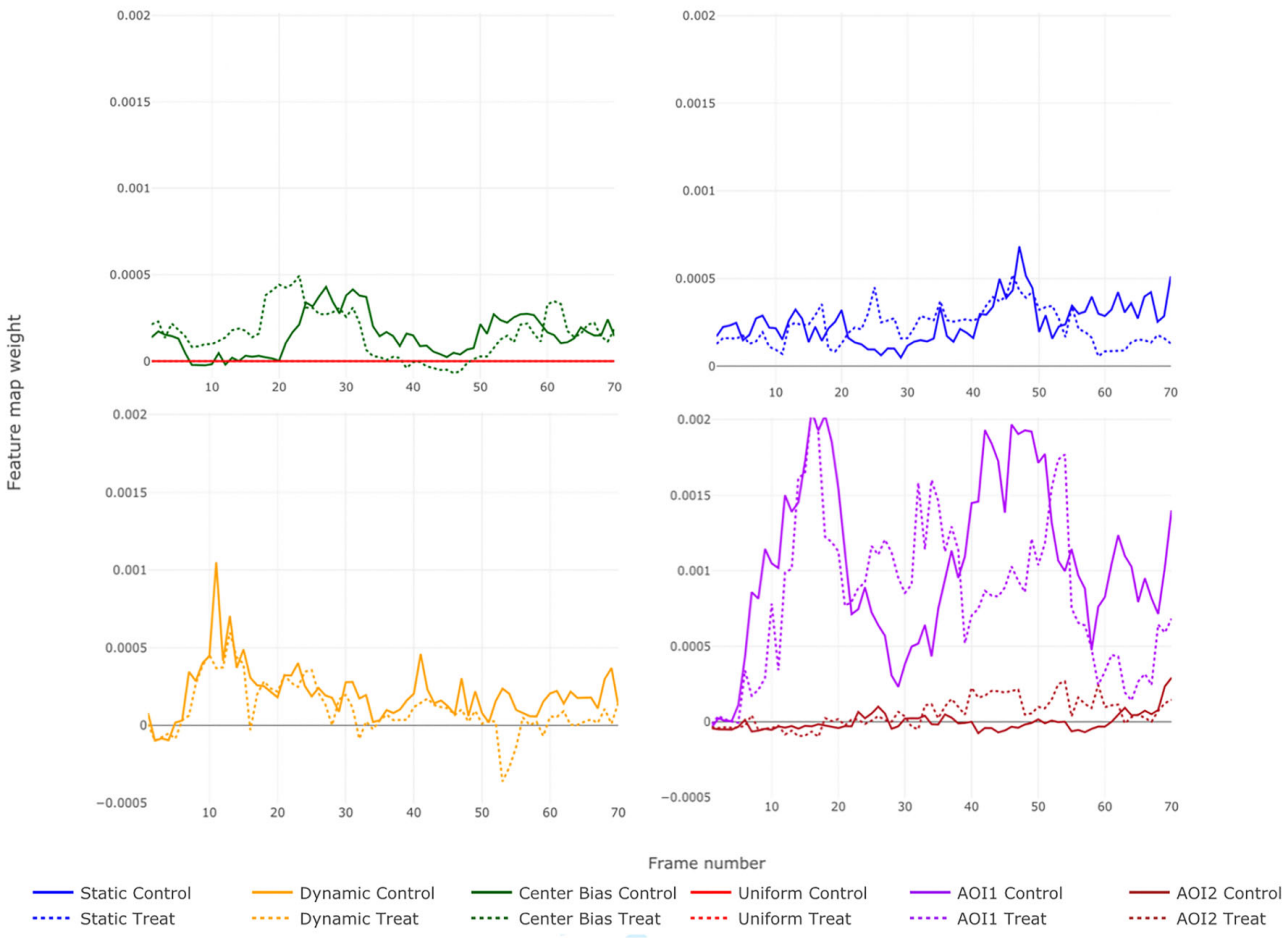
Estimated feature map weights for the first 70 frames in the two-group model for the clarity in multiple plots. *Dashed lines* lines stand for feature map weights in the treatment group and solid for the control group

The *R*^2^ values in the group model for the stimulus vary over the entire duration of the stimulus between values close to zero and 0.5, with most frames showing *R*^2^ values between 0.1 and 0.4. Apart from the very low *R*^2^ values of the models of the first frames, no temporal influence on the *R*^2^ values can be seen. The comparison of the results of the model with a LASSO penalty to the results of a least squares approach shows that there are no notable differences in the results (see Fig. 10) which means that all features in the model play an essential role in explaining the gaze behavior. Figure 6 shows the non-normalized RI curves or feature map weights *β*_*U*_,...,*β*_*AOI*2_ for the control group in transparent colors and *β*_*U*_ + *β*_*U*,*G*_,...,*β*_*AOI*2_ + *β*_*AOI*2,*G*_ for the treatment group. The weights here are illustrated in a non-normalized form, since the densities of the two groups were not scaled equally and differ in particular in their maximum. When interpreting such results, it should always be taken into account that inaccuracies in the eye tracker may lead to a fixation being incorrectly assigned to a feature. For the AoIs and the center bias as well as for the dynamic saliency this problem should be rather negligible, since these features cover comparatively large and dense areas of the stimulus. The influence of these inaccuracies can be greater for the static saliency, which in some cases highlights very fine contours (see Fig. 1). The curves are descriptive in nature and do not indicate significant influences of some features on the fixations or differences between the groups. In both groups, however, the dynamic AoI (AoI1) seems to have a higher weighting than the other features, which suggests that gaze behavior is strongly driven by the stimulus content. Nevertheless, the dynamic AoI is moving in a linear way with no changes in velocity or directions, so for the participants it is very easy to predict the development of the depicted movement, which in turn frees them to explore the rest of the scene. Overall, the curves in both groups run at a similarly high level. The curves indicate that the groups do not react to image elements represented by the feature maps at exactly the same time, but with a time lag. This behavior can be seen for example in the center bias curves (green). Also, the curves of the dynamic AoI (AoI1) indicate that the groups do not always focus on the car at the same time. Since we use the *L*_1_ penalty in our model, feature map weights, which are not relevant for the prediction of fixations, would get a value of zero, which is not the case here except for the uniform map, which serves as a control instance and should therefore be zero. It can be concluded that all feature maps in our model, the bottom-up feature center bias as well as the top-down features, seem to be relevant for the prediction of fixations.

**Fig. 7:**
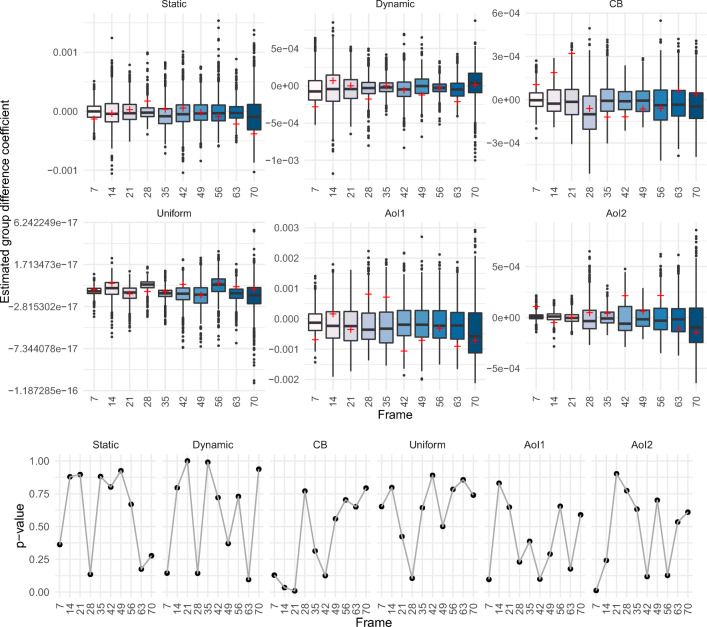
Boxplots showing the estimated coefficients of the group differences from 1000 permutations in all feature maps in our proposed model (1) for a selection of equidistant frames of the stimulus *car cornfield* (*top*). *Red crosses* indicate the estimated coefficient of the true groups. Corresponding *p* values for each feature map and frame (*bottom*)

**Fig. 8:**
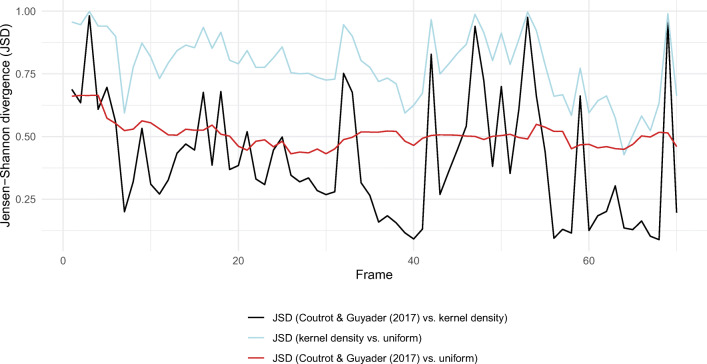
Jensen–Shannon divergence (JSD) between the kernel density estimate (KDE) in Coutrot and Guyader (2017) (bandwidth 1 degree of visual angle) and the KDE with a bandwidth selection via bivariate least squares cross-validation as well as the JSD between the kernel densities and the uniform distribution for the stimulus *car cornfield*

**Fig. 9:**
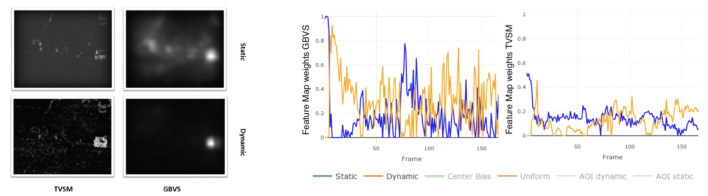
Illustration TVSM and GBVS saliency maps for one frame of the stimulus *car cornfield* (*left*) and resulting feature map weights (static and dynamic saliency) for GBVS and TVSM saliency maps (*right*)

**Fig. 10:**
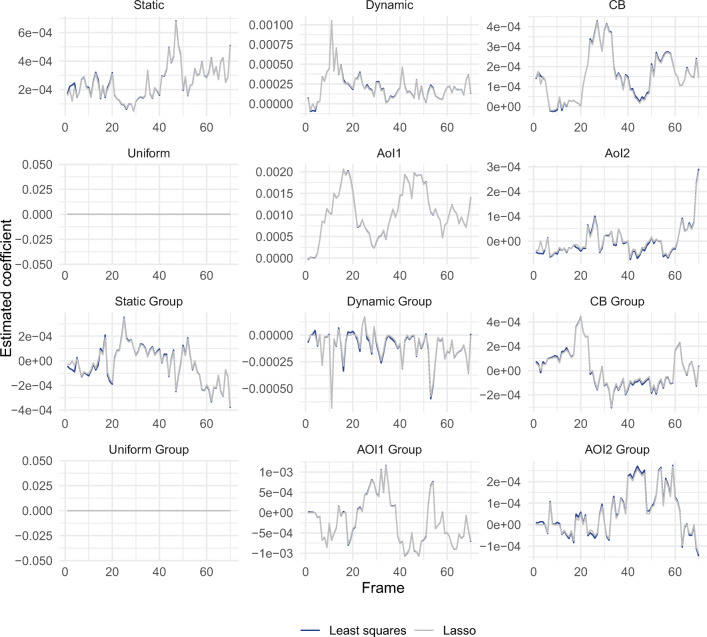
RI curves for the least-squares approach (*blue*) and for the lasso penalty (*gray*)

We have performed permutation tests based on an equidistant sequence of ten frames (for runtime reasons) to make statements about significant differences between the groups for each frame. The following Fig. 7 shows the boxplots of all observed coefficients of group differences $\hat {\beta }_{U,G},...,$ $\hat {\beta }_{AOI2,G}$ in *P* = 1000 permutations and the corresponding *p* values. The observed coefficients are highlighted with a red cross. If the red cross is located inside the box, the regression coefficient hardly differs from the coefficients of a random group assignment and this indicates that there exists no difference in gaze behavior between the two groups in this frame. Conversely, observations located outside of the box represent significant group differences. The results we obtain reflect what the relative importance curves indicate.

For example, in frame 42 for both the static AoI (AoI2) and the dynamic AoI (AoI1) and also for the center bias the red crosses are outside of the box as one would expect when looking at the RI curves. For frame 21, on the other hand, no significant difference can be detected in both AoIs, which is also indicated by the curves. The fact that the AoI1 boxes are not exactly centered around zero and are also very large overall shows that in the dynamic AoI, the gaze behavior also varies more among individuals in general. Nevertheless, the difference between the two groups examined here is particularly noticeable.

**Fig. 11:**
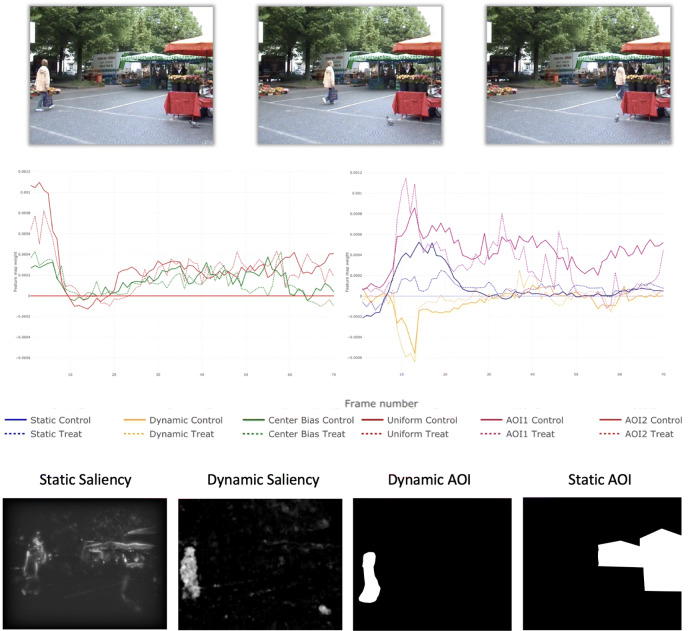
Relative importance curves of the stimulus walking market with the lady as dynamic AoI (AOI1) and the market stalls as static AoI (AOI2). There is also a pigeon walking through the video (starting at the lower right corner), which is not modeled as a separate AoI

**Fig. 12:**
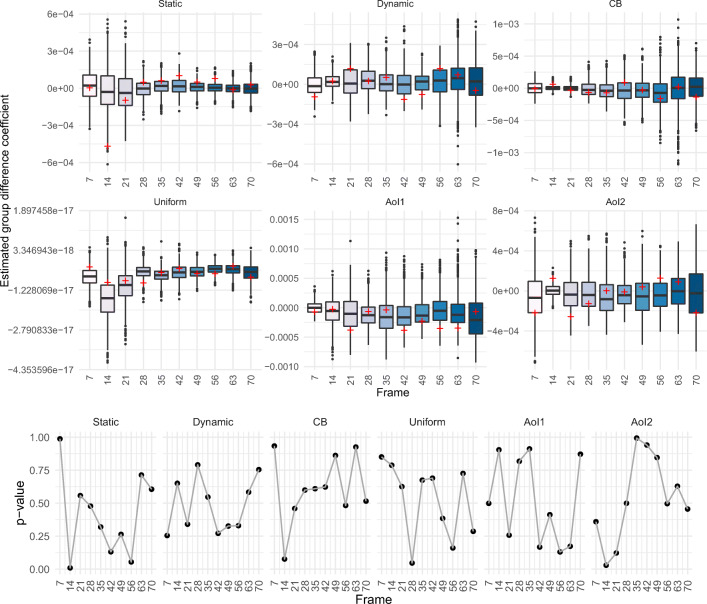
Boxplots showing the estimated coefficients of the group differences from 1000 permutations in all feature maps in our proposed model (1) for a selection of equidistant frames of the stimulus *walking* *market* *(top*). *Red crosses* indicate the estimated coefficient of the true groups. Corresponding *p* values for each feature map and frame (*bottom*)

We again use model (1) and the same groups to analyze a further stimulus *walking market*, which includes a market stall as an endpoint and a lady as a moving object moving towards the market stall. This stimulus also contains other possible areas of interest such as a pigeon walking through the image, which are not included as individual features in the model, but are covered by the static and dynamic saliency. Again, we find that the dynamic AoI (AoI1), i.e., the lady, gains the highest weight in both groups. In contrast to the previous stimulus, the static AoI (AoI2) has high weights at the beginning, which can be explained by the numerous elements in the picture. Example frames of the stimulus and the resulting RI curves are shown in Fig. 11 and results from permutation tests in Fig. 12.

**Fig. 13:**
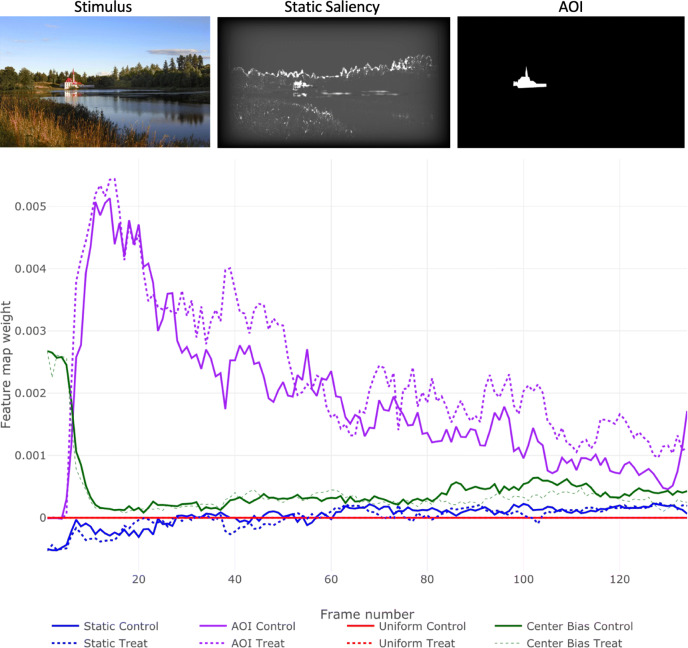
Group comparison of gaze behavior between a group of *N*_*T*_ = 55 (after matching) architecture and civil engineering students (treatment) and a group of *N*_*C*_ = 55 (after matching) linguistic students (control) over time for a static stimulus and one AoI (church)

**Fig. 14:**
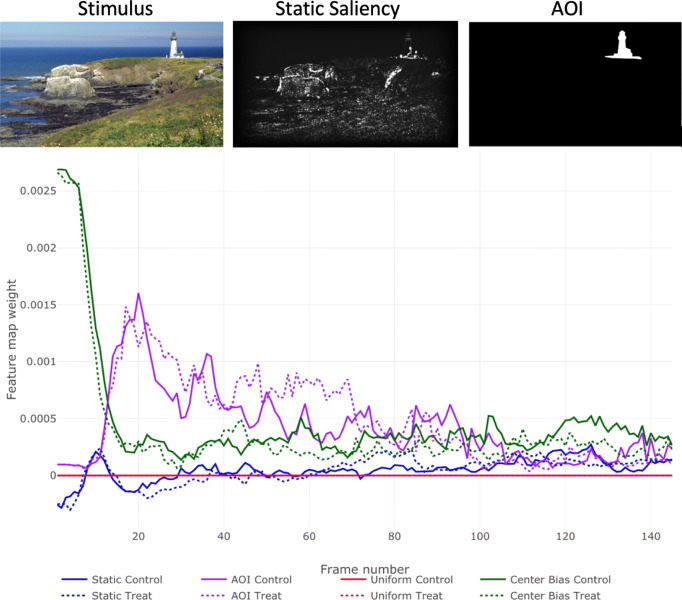
Group comparison of gaze behavior between a group of *N*_*T*_ = 55 (after matching) architecture and civil engineering students (treatment) and a group of *N*_*C*_ = 55 (after matching) linguistic students (control) over time for a static stimulus and one AoI (lighthouse)

Depending on the experimental context, additional AoI maps could be added, say to model objects competing for attention. Following Coutrot and Guyader (2017), we think that both saliency maps are advisable to include in calculations, but the underlying LASSO regression modeling framework continues to work when one or both saliency maps are removed from Eq. 1, changing model interpretation when doing so. To illustrate the generalizability of our approach, we include the evaluation of static stimuli in which the dynamic AoI and the dynamic saliency in model (1) are omitted (see Figs. 13 and 14). We expect that the method can also be applied to video stimuli with camera panning, since neither kernel density estimation, AoI maps nor saliency map calculations rely on static scenes.

## Conclusions

This article provides a multi-group extension of a visual saliency model for dynamic stimuli by Coutrot and Guyader (2017). This allows to compare two or more natural or experimental groups in terms of the relative importance (RI) of visual features. Standardized RI plots provide an interpretable summary. The practical application of the method shows that the RI curves have similar shape in both groups, despite the more explorative gaze behavior in the treatment group. The method thus represents a group comparison tool which is robust against possible intentional changes in gaze behavior and investigates differences in highly automated and subconscious gaze behavior. In contrast to dynamic models on the level of individuals, gaze behavior is first aggregated groupwise for each frame. Hence, model coefficients and especially RI have to be interpreted as parameters of the groups gaze behavior distribution. In general, it is not possible to make predictions for individuals. In principle, the linear model framework in the background is extensible to further covariates. However, we caution that the gaze distribution needs to be estimable by kernel densities or similar approaches, which breaks down when too few individuals are available.

We demonstrate that the method also works for fewer features and for static stimuli. The method and the provided code are applicable to other natural groups and video stimuli without camera panning without any major changes. The single steps of model construction can be individually adapted and should be reflected with regard to the stimulus material.

## Footnotes

1 https://github.com/marastadler/Lasso_eyeposition
